# The Role of Saliency in Learning First Words

**DOI:** 10.3389/fpsyg.2019.01150

**Published:** 2019-05-15

**Authors:** Eugenia Wildt, Katharina J. Rohlfing, Ingrid Scharlau

**Affiliations:** Faculty of Arts and Humanities, Paderborn University, Paderborn, Germany

**Keywords:** word learning, saliency, relevance, joint attention, joint action

## Abstract

In word learning, one key accomplishment is the reference, that is, the linking of a word to its referent. According to classical theories, the term *reference* captures a mental event: A person uses a word to mentally recall a concept of an entity (an object or event) in order to bring it into the mental focus of an interaction. The developmental literature proposes different approaches regarding how children accomplish this link. Although researchers agree that multiple processes (within and across phonological, lexical, and semantic areas) are responsible for word learning, recent research has highlighted the role of saliency and perception as crucial factors in the early phases of word learning. Generally speaking, whereas some approaches to solving the reference problem attribute a greater role to the referent’s properties being salient, others emphasize the social context that is needed to select the appropriate referent. In this review, we aim to systematize terminology and propose that the reason why assessments of the impact of saliency on word learning are controversial is that definitions of the term *saliency* reveal different weightings of the importance that either perceptual or social stimuli have for the learning process. We propose that defining early word learning in terms of paying attention to salient stimuli is too narrow. Instead, we emphasize that a new link between a word and its referent will succeed if a stimulus is *relevant* for the child.

## Introduction

Studies vary in their suggestions regarding how links emerge between words and referents: Some explanations build on research in the field of the psychology of perception (see Table 1) confirming that saliency (or salience) can capture attention quite effectively. Bottom-up saliency (Latin *salire*: to jump) is by definition a property in objects that makes them stand (or jump) out of the surrounding context. For instance, a red jacket stands out in the context of a crowd of black jackets (Itti and Koch, 2001: 194). Besides visual features such as color, luminance, orientation, motion, or size, auditory properties such as loudness, pitch, or spectral shape can attract attention in a mainly bottom-up way (e.g., Treue, 2003). Whereas approaches taking advantage of saliency propose that during word learning, salient properties capture and sustain infants’ attention for an object during the time in which the referent is being labeled, other explanations emphasize social interaction and its goals and how reference is established for this among partners. Our aim is to systematize the use of terms and sensitize the reader to the difference between *saliency* and *relevance*. We argue that relevance is achieved by embedding the child’s perspective into a social environment—that is, a history of joint actions. In the following, we first review studies that focus on the role of saliency for word learning.

**Table 1:** State of research on saliency in early infancy.

Topic of research on salient properties	Results	References
Patterned vs. Monochromic stimuli	5-day-olds prefer black-and-white patterns over monochromic surfaces	Fantz, 1963
Development of color preference: Chromatic vs. Achromatic	Newborns and 3-month-olds prefer chromatic over achromatic stimuli; 3-month-olds prefer red and yellow over blue and green	Adams, 1987
Dynamic vs. Static stimuli	5-month-olds prefer moving over static female faces	Wilcox and Clayton, 1968
Dynamic objects in combination with chromatic stimuli	14-week-olds’ attention to moving stimuli depends on color of target object: When visual field is a mixture of red and green stimuli, infants have difficulties in attending to a green target object that starts to move; when a red object starts to move, infants readily switch their attention to it	Nagata and Dannemiller, 1996
Change of color vs. Change of rotation speed	9-month-olds notice a salient difference in color change, but not in a change of rotation speed	Kaldy and Blaser, 2013
Development of recognizing changes in salient features	6-month-olds require larger differences in color, shape, or luminance to identify an object change compared to 9-month-olds	Kaldy and Blaser, 2009

## Mechanistic View on Word Learning

The mechanistic view (e.g., Smith, 1995; Smith et al., 1996; Samuelson and Smith, 1998; Spencer et al., 2011; see also Plunkett, 1997, for a review) proposes that general mechanisms such as memory and attention drive the word learning process. The child’s task is to link the heard phonological form with an entity (an object or event) that he or she is attending to visually. Supporting studies have boiled word learning down to essentially two problems: First, the learning environment is ambiguous [a problem already identified by Quine (1960)], because it offers many potential referents and distractors (Trueswell et al., 2016). Second, even if infants identify the referent correctly, they have difficulties in sustaining their visual focus on the referent because of their still maturing attentional skills (Yu and Smith, 2016). Thus, the mechanistic view centers on the question how infants solve the ambiguity problem and sustain their attention on a referent in order to successfully establish a new word–object link.

One approach considers saliency to be a precondition for recruiting infant’s attention for an object, which in turn, if temporally synchronized with labeling, will establish a link between a word and its referent in the infant’s memory (Gogate and Bahrick, 1998). We shall collectively name these studies *associationist* (Hollich et al., 2000: 12), although they operationalize saliency differently (see below). A related position assigns a greater role to child’s growing experience by postulating *constraints* and *principles* of learning and is presented in more detail below.

## Saliency—A Property in Objects

The associationist account postulates that infants solve the ambiguity problem because of their preference for salient objects or because they assume that adults will label that object which is the most interesting from the infant’s point of view. This account implies that for early word learning, infants rely more on perceptual saliency than on social stimuli (Moore et al., 1999; Hollich et al., 2000). This was demonstrated by Pruden et al. (2006) who operationalized social stimuli as the gaze of the experimenter and compared its effect with that of perceptual saliency: In a “coincidental condition” (p. 269), the experimenter gazed at and simultaneously named a salient object; in a “conflict condition,” the experimenter gazed at and labeled a “boring” object while a salient object was present. Infants in the coincidental condition spent more time looking toward the salient object, indicating that they mapped the new word onto the intended referent. The conflict group also looked longer at the salient distractor, indicating that the label was mismatched with the salient object. The authors viewed their results as evidence that young infants weight object saliency higher than social cues in their word learning process. Only in the course of development, do infants “move from learning words associatively to learning words based on the social cues a speaker emits” (p. 278). However, Golinkoff and Hirsh-Pasek (2006) noted that it is still not known how child development reveals a qualitative change toward weighting social cues more than perceptual cues.

Crucially, even though Pruden et al. (2006) did not define the term *saliency* explicitly in their study, they did vary it methodologically in terms of attention-grabbing properties—that is, in terms of objects that recruit and hold infants’ visual attention: The salient stimuli were brightly colored and could either make a noise or move, and they were paired with boring objects (dull color, neither motion nor noise). These properties are consistent with the psychological view on bottom-up saliency.^1^ Other studies extended this notion: For example, an object’s saliency increases if it is larger (Smith et al., 1996; Pereira et al., 2014) or more centered in the infant’s visual field in comparison to other toys, rotates on a turntable (Moore et al., 1999), moves (Werker et al., 1998; Houston-Price et al., 2005, 2006), or is illuminated (Axelsson et al., 2012). It should be noted that even though infants attend to a moving object during training, they exclude it as referent at test if its movement is not consistent (Houston-Price et al., 2006). In this way, saliency is regarded as a “bottom-up sensory input that is clean” (Yu and Smith, 2012: 258), meaning that only one object dominates the visual view. In natural environments, parents can facilitate their infants’ word learning if they establish such visually optimal moments by bringing the target object more to the fore. Furthermore, infants often create situations on their own in which referential ambiguity is low (e.g., during toy play by exploring one object at a time). Object naming in these moments is associated positively with word learning (Yu and Smith, 2012; Pereira et al., 2014).

With regard to the mechanistic view, results indicate that word learning is driven by general processes of attention and memory that are recruited via attention-grabbing features in objects. This saliency effect can be explained by cognitive learning mechanisms being facilitated by the diminishment of competitors and the unambiguous determination of the referent (Axelsson et al., 2012).

## Saliency—A Property Generated by the Perceiver

Without opposing the role of perception, another perspective highlights infants’ experience with word learning episodes. Being exposed to referents and their labels, the cognitive demand, namely to map the label onto some features of the referent, gives rise to necessary cognitive operations. Accordingly, children make use of *constraints* and *principles* (Markman, 1994) that narrow down referent selection. From this position, the salient property in objects derives from the knowledge (and experience with the labeling task) of the perceiver. Such constraints and principles as the *whole-object assumption* (Markman and Wachtel, 1988; Woodward, 1992) or *mutual exclusivity* (Markman and Hutchinson, 1984; Clark, 1987; Markman, 1989; Merriman et al., 1989; Waxman and Kosowski, 1990; Golinkoff et al., 1992, 1994) are of different natures. While the whole-object assumption guides infants to the object’s features that they need to select for labeling, *mutual exclusivity* describes a bias in infants that prevents them from linking new words with already named objects because of the underlying assumption that objects can bear only one label (Markman, 1989, 1990). Indications of mutual exclusivity have been observed in infants as young as 10 months (Mather and Plunkett, 2010), but the cognitive basis of such a bias remains disputed: Is mutual exclusivity based on the knowledge of familiar versus novel labels for objects (e.g., Mervis and Bertrand, 1994) or rather the knowledge of an object’s novelty (e.g., Merriman et al., 1995; Mather and Plunkett, 2010; Horst et al., 2011)? At this point, we would like to note that we limit our considerations here to studies investigating the novelty bias.

To determine this bias, Mather and Plunkett (2012) presented 22-month-old infants with two stimuli: Both were name-unknown, but only one of them was truly novel. The children had become familiar with the other object through pre-exposure to it. Results showed that after having heard a new word, infants’ attention, in the form of looking time, increased more toward the novel object compared to the familiar one. The authors concluded that mutual exclusivity is a novelty-based mechanism, because it seems to be cognitively easier for infants to search for a perceptually novel object in the environment rather than retrieving all familiar object names. Hence, when presented with a novel word, the most novel object will appear to be the most salient one to an infant, thereby facilitating the mapping process. The novelty bias is a good example for a learning constraint, although we do not suggest that all constraints and principles are attributable to saliency in the same way as novelty.

One aspect is crucial to this position: Rather than being a salient object emerging through bottom-up attention, saliency is generated by the perceiver through top-down processes (Connor et al., 2004). When facing the ambiguity problem, infants rely on their prior knowledge to identify whether not only words but also objects are actually novel. The ability to hear a new label and to map it onto a novel, name-unknown object, characterizes infants as active learners in their environment. This perspective holds that word learning is a cognitive process that also affords a top-down mechanism (including past memory) in the perceiver.

## Social-Pragmatic and Interactionist View on Word Learning

Whereas in the mechanistic view, word learning is dependent on perceptual and attentional constraints, social-pragmatic theories (e.g., Bruner, 1983; Tomasello, 2000; Csibra and Gergely, 2006) claim that from early on, infants are sensitive to social cues (see Table 2).

**Table 2:** State of research on social sensitivity in infancy.

Topic of research	Results	References
Visual preferences	Newborns prefer to look at face-like compared to non-face-like stimuli; preference for faces declines during second month of life	Fantz, 1963; Johnson et al., 1991
	Infants discriminate their mother’s faces from foreign ones	Field et al., 1984; Bushneil et al., 1989; Pascalis et al., 1995
Listening preferences	Infants prefer listening to human speech compared to rhesus calls, as well as speech compared to non-speech sounds; this listening bias for human speech is innate	Vouloumanos and Werker, 2004; Vouloumanos and Werker, 2007; Vouloumanos et al., 2010
	From birth, infants prefer listening to infant-directed compared to adult-directed speech	Fernald, 1985; Cooper and Aslin, 1990; Pegg et al., 1992
Following others’ gaze direction	Infants prefer human faces and are sensitive to direct eye contact from birth	Farroni et al., 2002
	6- to 18 month-olds follow others’ gaze direction if the referent is in their visual field	Butterworth and Cochran, 1980
	Three mechanisms serve joint attention in the first 18 months	Butterworth and Jarrett, 1991
	Infants do not reliably follow others’ gaze direction for joint visual attention before the age of 18 months	Moore and Corkum, 1998
	Differences in the capacity of gaze following at 6 months of age relate to vocabulary development	Morales et al., 1998
Responses to others’ gestures	12-month-olds but not 9-month-olds follow gestures to targets behind them	Deák et al., 2000; Flom et al., 2004
	4.5-month-olds follow dynamic, but not static pointing gestures	Rohlfing et al., 2012
Responses to aspects in social interactions	2-month-olds show organized facial expressions that are responsive to maternal communication	Trevarthen, 1985
	Infants are more responsive to mothers in live compared to replayed videotape sequences	Murray and Trevarthen, 1986
	Right from early on, infants are socialized as participants in interactions	de Leoìn, 2000
	Early vocal exchange between infant and mother has a turn-taking format	Masataka, 2003; Gratier et al., 2015
	Infants are sensitive to other’s contingent actions	Kaye, 1977; Jaffe et al., 2001; Striano et al., 2005
Understanding social events	14-month-olds show understanding of social intentions; they imitate intentional actions more than accidental actions	Carpenter et al., 1998

Even though both social-pragmatic and interactionist perspectives agree that word learning is an inherently social process, they differ in how referents become salient during social interaction. We shall elaborate on this difference in the following.

## Saliency—A Property Emerging From Social Perception

One line of social-pragmatic studies (Tomasello and Akhtar, 1995; Baldwin et al., 1996; Carpenter et al., 1998) proposes that because infants are especially sensitive to objects that adults single out via *ostensive means* (eye gaze, gestures, or emotions), these objects become salient and can then be linked with new words (Akhtar et al., 1996; Csibra, 2010; Axelsson et al., 2012). In a study with 24-month-olds, Horst and Samuelson (2008) used ostensive naming (i.e., addressing the child directly, holding up the target object, and pointing at it) as a form of specific social behavior that singled out the target object and reduced competition from distractors. Compared to a condition in which a non-ostensive naming was provided, retention of new words and thus long-term learning was observed only in the ostensive naming condition. It can therefore be concluded that the use of social cues not only facilitates infants’ encoding but, more crucially, induces their long-term processing.

Recent research on infants’ reactions to attention-directing social cues supports the assumption that caregivers’ actions do not just facilitate shared attention, but might also aid learning. For example, Deák et al. (2018) found that five properties are salient from a perceiver’s perspective: gaze shift, pointing gestures, speech, object sounds, and object manipulation. They argued, for example, that pointing gestures are salient due to the sweeping motion of the arm and hand (see also Rohlfing et al., 2012). Their results indicated that when used within a dyadic toy play interaction, 3- to 11-month-olds were sensitive to all five different caregiver social cues, with object manipulation being the most effective (see also Yu and Smith, 2012).

Effects of saliency as a property of social perception can be explained by natural pedagogy (Csibra and Gergely, 2009). This stipulates that from early on (and possibly even from birth, Csibra, 2010), infants are sensitive to ostensive signals. Because they understand the referential nature of communicative signals (Gliga and Csibra, 2009), they follow them to indicate the word reference (Baldwin, 1993; Baldwin et al., 1996).

In sum, this line of social-pragmatic research reveals that social cues evoke a high level of attention in the perceivers and guide them toward objects relevant for the interaction. Furthermore, research on the attention of adults makes a similar distinction between top-down attention, which is guided by goals, knowledge, or expectations; and bottom-up attention, driven by stimulus contrast or salience (e.g., Folk et al., 1992). This might point to a more general mechanism that drives word learning as well as visual attention.

## Away From Saliency—Relevance Emerging in Interactions

In comparison to the line of research mentioned above, interactionist theory turns away from the view that infants build references by observing salient entities. Instead, a successful object–referent association is attributed to infants’ *engagement* in an interaction toward a joint goal. Proponents of this approach emphasize the importance of a pragmatic frame that is established by repetitive participation in an ongoing social event leading to interactional experience and particular communicative acts (in the form of joint attention) that serve word learning (Bruner, 1983; Rohlfing et al., 2016).

Along these lines, Wildt and Rohlfing (2018) investigated word learning in 10-month-olds by directly comparing the associationist, the social-pragmatic, and the interactionist approaches. A stimulus set consisting of one perceptually highly salient and one less salient object was presented on a screen within an intermodal preferential looking paradigm (IPLP). Infants’ visual attention was estimated by an eye tracker. Infants’ interest for the stimulus set was assessed as the baseline. This confirmed that all infants preferred the salient stimulus. After that, all infants participated in an interaction: While both stimuli lay side by side on a table, the experimenter gazed at the “boring” stimulus, repeatedly demonstrated its function, and labeled it ostensively. After ostensive naming, infants were divided into two groups and encouraged to play with the less salient object. One group explored the object’s function on their own without any further input from the experimenter; the other group explored it in an interaction. The specific manipulation in the second group was for the experimenter to provide the infants with support in manipulating the boring object to achieve a joint goal. Here, we were contrasting the impact of joint attention with the impact of joint action on word learning. Our prediction was that infants would match a new word to the boring object because it was demonstrated as relevant for the interaction. Even though the salient object was always in the infants’ field of view, subsequent visual attention provided evidence that infants’ engagement was crucial to establish the reference. These results contradict the associationist approach and demonstrate that young infants’ early attention is not driven merely by perceptually salient objects. In addition, the results on the contrast between the joint action and the joint attention condition reveal that establishing joint attention is not sufficient to develop a word–referent link for a boring object, because infants in this condition still preferred the salient object as at baseline. In contrast, an interaction in which infants participated had a stronger effect on infants’ perception, because they no longer showed a preference for the salient object. In this vein, while the contribution of pointing behavior to later language development is recognized, recent studies reveal that this relationship might rely on pointing to interaction-relevant entities (Białek et al., 2018). Again, we want to point out a possible parallel to theories of attention in adults that are as yet unexplored: Selection-for-action approaches assume that attentional selection mainly serves action control (e.g., Neumann, 1987). Even though there is no particular reference to interactions in these approaches, interactions and actions may share the crucial property that action capacities are limited. Further research needs to clarify whether saliency emerging from interactions accords with theoretical approaches on selection for action in adults.

Taken together, the interactionist approach differs in its perspective on word learning by claiming that infants need not only perceptual or social saliency but also an active experience in an interaction such as achieving a relevant function or a joint goal. Hence, a referent becomes relevant by “charging” it with a rich meaning from actions that have taken place within a social interaction (Nomikou et al., 2016) and that draw on what the child knows (Bloom et al., 1993) rather than merely being salient in a joint attention or visual attention scenario.

## Discussion

**Figure 1:**
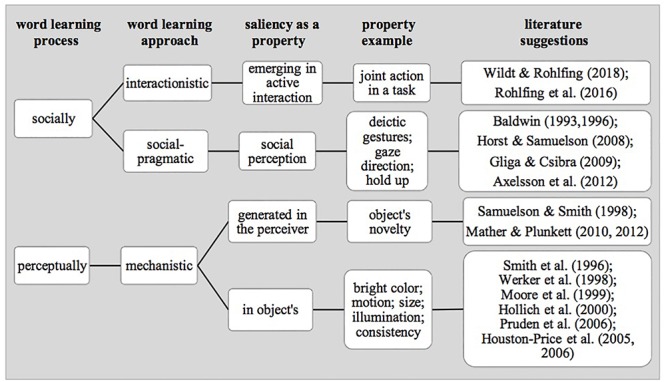
State of research on saliency (emerging from perceptual biases, social cues, or interactions) in the context of early word learning.

We surveyed how the term *saliency* is used in three theoretical approaches to the study of word learning. Systematizing its uses, we registered that they differ in terms of the prioritization of the non-social versus the social information that is recruited as a cue in the process of word learning (see Figure 1).

The difference in mechanistic approaches lies in the internalization of saliency: Whereas from the associationist view, bottom-up mechanisms drive infants’ attention toward attention-grabbing properties, the approach using constraints and principles suggests that a perceiver’s top-down driven attention is based on past knowledge. The recruitment of the experience in perceiving an object is what makes it salient; or to put it in more appropriate terms: relevant.

This review addressed two other approaches that consider saliency to be socially driven. This means that infants are sensitive to social cues from early on, and that word learning is inherently social. Whereas in one line of social-pragmatic studies, saliency is attributed to infants’ social perception and their responsivity to cues such as eye gaze and pointing or ostensive labeling, a second interactionist line of studies claims that the pragmatic frame of a joint action is needed for the infant to recognize the relevance of an object for the joint goal of this interaction. In this latter view, a word–object link becomes charged with interwoven words and actions. Therefore, infants must be additionally embedded in joint actions and achieve a joint goal to see the purpose of a word and to involve their memory processes.

Taken together, it becomes clear that a unified theory is lacking. As a first step, we propose that future research should focus more on the term *relevance* rather than *saliency*, because it better encompasses the (social) context in which infants attend to objects.

## Author Contributions

EW and KR developed the framework and wrote the mini-review. This paper benefits from the expertise of IS in visual saliency for information processing.

## Conflict of Interest Statement

The authors declare that the research was conducted in the absence of any commercial or financial relationships that could be construed as a potential conflict of interest.

## Footnotes

1 Note that the notion of pure bottom-up capture that depends only on stimulus features and not on current goals is a contested issue in experimental psychology; the conditions under which irrelevant salient stimuli capture attention are still not fully understood.
